# Protein Restriction in the Peri-Pubertal Period Induces Autonomic Dysfunction and Cardiac and Vascular Structural Changes in Adult Rats

**DOI:** 10.3389/fphys.2022.840179

**Published:** 2022-04-28

**Authors:** Anna Rebeka Oliveira Ferreira, Maiara Vanusa Guedes Ribeiro, Maria Natalia Chimirri Peres, Silvano Piovan, Géssica Dutra Gonçalves, Lucas Paulo Jacinto Saavedra, Juliana Nunes de Lima Martins, Marcos Divino Ferreira Junior, Keilah Valeria Naves Cavalcante, Gabriel kian Guimarães Lopes, Mariane Carneiro, Douglas Lopes Almeida, Rodrigo Mello Gomes, Jurandir Fernando Comar, James Andrew Armitage, Paulo Cezar de Freitas Mathias, Kesia Palma-Rigo

**Affiliations:** ^1^ Laboratory of Secretion Cell Biology, Department of Biotechnology, Genetics and Cell Biology, State University of Maringa, Maringa, Brazil; ^2^ Laboratory of Liver Metabolism and Radioisotopes, Department of Biochemistry, State University of Maringa, Maringa, Brazil; ^3^ Laboratory of Endocrine Physiology and Metabolism, Department of Physiological Sciences, Federal University of Goias, Goiania, Brazil; ^4^ School of Medicine, Deakin University, Waurn Ponds, VIC, Australia; ^5^ Adventist College of Parana, Ivatuba, Brazil

**Keywords:** low protein diet, hypertension, autonomic nervous system, developmental origins of health and disease, peri-puberty

## Abstract

Perturbations to nutrition during critical periods are associated with changes in embryonic, fetal or postnatal developmental patterns that may render the offspring more likely to develop cardiovascular disease in later life. The aim of this study was to evaluate whether autonomic nervous system imbalance underpins in the long-term hypertension induced by dietary protein restriction during peri-pubertal period. Male Wistar rats were assigned to groups fed with a low protein (4% protein, LP) or control diet (20.5% protein; NP) during peri-puberty, from post-natal day (PN) 30 until PN60, and then all were returned to a normal protein diet until evaluation of cardiovascular and autonomic function at PN120. LP rats showed long-term increased mean arterial pressure (*p* = 0.002) and sympathetic arousal; increased power of the low frequency (LF) band of the arterial pressure spectral (*p* = 0.080) compared with NP animals. The depressor response to the ganglion blocker hexamethonium was increased in LP compared with control animals (*p* = 0.006). Pulse interval variability showed an increase in the LF band and LF/HF ratio (*p* = 0.062 and *p* = 0.048) in LP animals. The cardiac response to atenolol and/or methylatropine and the baroreflex sensitivity were similar between groups. LP animals showed ventricular hypertrophy (*p* = 0.044) and increased interstitial fibrosis (*p* = 0.028) compared with controls. Reduced protein carbonyls (PC) (*p* = 0.030) and catalase activity (*p* = 0.001) were observed in hearts from LP animals compared with control. In the brainstem, the levels of PC (*p* = 0.002) and the activity of superoxide dismutase and catalase (*p* = 0.044 and *p* = 0.012) were reduced in LP animals, while the levels of GSH and total glutathione were higher (*p* = 0.039 and *p* = 0.038) compared with NP animals. Protein restriction during peri-pubertal period leads to hypertension later in life accompanied by sustained sympathetic arousal, which may be associated with a disorganization of brain and cardiac redox state and structural cardiac alteration.

## 1 Introduction

The World Health Organization (WHO) estimated that there are 1.28 billion hypertensive people in the world predisposing this population to stroke, myocardial infarction, heart failure and renal failure (WHO, 2021b). In 2019, 17.9 million people died from cardiovascular disease, with over three quarters of these individuals being based in low- and middle-income countries (WHO, 2021a). The projection for 2030 is that approximately 23.3 million people will die from cardiovascular disease across the globe, reinforcing the fact that hypertension is a public health problem associated with vast social and economic consequences (Mathers and Loncar, 2006).

There is now clear evidence that environmental insults, including dietary restriction or imbalance, during sensitive windows of development increases the susceptibility to noncommunicable chronic diseases in adulthood, including hypertension (Barker et al., 2002; Barker, 2004). This process, termed the Developmental Origins of Health and Disease, or developmental programming is not new, however the mechanisms underpinning the programming of hypertension are still being elucidated. Experimental programming models of malnutrition or nutritional imbalance during the perinatal period show that arterial hypertension may be associated with increase in vascular sympathetic tone (Mizuno et al., 2013; Prior et al., 2014; Barros et al., 2015; Paulino-Silva and Costa-Silva, 2016; Lim et al., 2021) as well as redox disorganization in the brainstem (Ferreira et al., 2016; Barrand et al., 2017; Ferreira et al., 2019), and heart (Nascimento et al., 2014). Furthermore, the hypertension may trigger a cardiac compensatory response, leading to cardiomyocyte hypertrophy, and greater collagen deposition (Rossini et al., 2017; De Jong et al., 2018; Assalin et al., 2019).

Post-weaning until the end of adolescence has also been established as a critical window of development. We have previously demonstrated metabolic disfunction in adult animals exposed to dietary protein restriction during puberty/adolescence (De Oliveira et al., 2013; De Oliveira et al., 2018). Furthermore, experimental studies in models of malnutrition in post-weaning/peri-puberty indicate that hypertension (Loss et al., 2007; Penitente et al., 2007; Murca et al., 2012; Penitente et al., 2014) and vascular sympathetic hyperactivity (Martins et al., 2011) occur immediately after the exposure period, and these may be associated with a central glutamate disorganization (Rodrigues et al., 2012). However, the long-term cardiovascular dysfunction in this model has not yet been evaluated, pointing to a hole in the literature concerning the programming of hypertension induced by insults during adolescence or the peri-pubertal period. The hypothesis of this study is that exposure to a low-protein diet during the peri-pubertal period induces hypertension in adulthood which is dependent on autonomic nervous system dysfunction.

## 2 Methods

The experimental protocol was approved by the Research Ethics Committee for Animal Use and Experimentation at the State University of Maringa (protocol number 4833210519). Male Wistar rats were obtained from the Central Animal Facility of the State University of Maringa and kept in the Sectorial Vivarium of the Department of Biotechnology, Genetics and Cell Biology for adaptation for 5 days. Animals were housed three per cage (polypropylene cages with 40 × 34 × 17 cm) remaining under controlled temperature (22 ± 2°C) and photoperiod (07:00–19:00, light cycle) conditions, with *ad libtum* access to water and food.

At post-natal day (PN) 30 animals were assigned into two groups; those fed an isocaloric low-protein diet palletized (4% protein; LP) or a balanced commercial control diet (20.5% protein; Nuvital®, Curitiba/PR, Brazil; NP; Table 1) until PN60. After the dietary manipulation period, from PN60 until PN120, all animals were fed the same commercial control diet (20.5% protein; Nuvital®, Curitiba/PR, Brazil).

**TABLE 1:** Composition of the isocaloric low- and normal-protein diets.

Diet components	Normal-protein (20.5%)	Low-protein (4.0%)
Sucrose	12.72	20.00
Cornstarch	52.75	64.25
Casein (88% protein)	23.33	4.55
Mix of mineral salts	3.20	3.20
Mix of vitamins	1.60	1.60
Soybean oil	4.80	4.80
Fish oil	1.60	1.60
*Total (g)*	100.0	100.0

At PN120 both groups underwent evaluation of the cardiovascular and autonomic nervous systems; biochemical, food intake, body composition, histological evaluation; and biochemical assays of protein expression and oxidative stress.

### 2.1 Body Composition and Food Intake

Throughout the experimental period, food consumption was recorded three times a week. Weekly food intake was calculated according to the formula [absolute consumption of the week x 100/average weekly weight of the box]. Bodyweight was recorded on a weekly basis.

At PN 120 a cohort of animals was fasted overnight and then euthanized with a guillotine. Overnight fasting was performed in order to match metabolic conditions between the animals for subsequent biochemical analysis. Bodyweight was recorded, naso-anal length evaluated and fat depots (retroperitoneal, mesenteric) were dissected and weighed.

### 2.2 Biochemical Parameters

Total blood was collected, and the serum separated for the biochemical dosages. Glycemia was quantified by the glucose oxidase method by spectrophotometry (semi-automatic biochemical analyzer, BIO 200FL, Bio Plus®, São Paulo/SP, Brazil), using a commercial kit (Gold Analisa®, Belo Horizonte/MG, Brazil). Total cholesterol was measured by the colorimetric method of cholesterol oxidase and triglycerides were measured using the colorimetric method of glycerol-3-phosphate oxidase using commercial kits (Gold Analisa®, BeloHorizonte/MG, Brazil). Both readings were performed on spectrophotometry equipment (Semi-automatic biochemical analyzer, BIO200FL, Bio Plus®, São Paulo/SP, Brazil).

HDL-cholesterol was determined after precipitation of chylomicrons and low-density lipoproteins with a commercial kit (Gold Analisa®, BeloHorizonte/MG, Brazil) and subsequent determination of HDL-cholesterol using the method described above for the measurement of total cholesterol.

### 2.3 Arterial and Venous Catheterization

After anesthesia (ketamine-xylazine; 75 mg + 15 mg/kg, i.p.) two cannulae (P10 -Micro-Renathane connected to P50 cannulae (ClearTygon)) were inserted; one into the femoral artery, for monitoring of arterial pressure, and one in the femoral vein, for drug administration. At the end of surgeries, animals received anti-inflammatory analgesic (Meloxican, 0.4 mg/kg, i.v.) and antibiotic (Enrofloxacin 5 mg/kg, i.v.) cover, the lines were heparinized (250 units/ml) after arterial catheter implantation and animals kept in individual boxes for recovery (Barros et al., 2015; Paulino-Silva and Costa-Silva, 2016; Simas et al., 2018). After 24 h of recovery, the protocol for assessment of cardiovascular function and its autonomic modulation was carried out over a period of 3 days.

### 2.4 Arterial Pressure Recording

After 1 hour of room adaptation, baseline arterial pressure records were performed for 30 min on each of the 3 days of the experimental protocol. Following baseline recording, intravenous injections were performed according to the protocol described below. Experiments were carried out between 10:00 and 14:00 in conscious free-moving animals in order to avoid the influence of circadian variations on cardiovascular autonomic modulation and to obtain recordings that were not subject to a high degree of locomotor activity.

The arterial cannula was connected to a pressure transducer (MLT0670, ADInstruments, Dunedin, and New Zealand) which was connected to a recording system (Insight, Ribeirão Preto, Brazil) that uses the DI 158 System for signal acquisition and analog-to-digital signal conversion (WinDaq lite, DataQinstruments, United States of America). Recordings were sampled at 1000 Hz.

Data were pre-analyzed using specific Advanced CODAS software (Data Q instruments, United States of America, https://www.dataq.com/products/windaq/, paid software) to objectively identify stable periods of recording and exclusion of erratic signals. The baseline values of Systolic Arterial Pressure (SAP), Diastolic (DAP), Mean (MAP) and pulse interval (PI) were transferred to a spreadsheet for analysis (Microsoft Excel, Redmond, WA, EUA) over 10 min of recording under stable conditions. Heart rate (HR) was calculated from the pulse interval (ms), considering formula (60000/PI in ms) and the pulse pressure was calculated through the difference between SAP and DAP.

### 2.5 Cardiovascular Variability and Baroreflex Sensitivity

The estimation of autonomic impact on baseline AP and PI was evaluated in the frequency domain through the analysis of MAP and PI variability, using CardioSeries 2.4 Software (USP - Ribeirão Preto, http://www.danielpenteado.com/, freely available). The power spectra were calculated using a fast Fourier transform (FFT) as previously describe (Santos et al., 2012; Silva et al., 2015; Speretta et al., 2016). Briefly, Beat-by-beat series obtained from pulsatile arterial pressure recordings were converted to data points every 100 ms using cubic spline interpolation (10 Hz). The interpolated series were divided into half-overlapping sequential sets of 512 data points (51.2 s). Before calculation of the spectral power density, segments were visually inspected, and nonstationary data were not taken into consideration. To confirm that the visual inspection of the time series was properly performed, a Hanning window was used to attenuate side effects and all segments had the spectrum computed using a direct FFT algorithm for discrete time series. All segments were visually inspected for abnormal spectra. The low frequency band of the MAP (LF-MAP: 0.2–0.75 Hz of the spectrum) was used as an estimate of vascular sympathetic activity whilst the low frequency band of PI (LF-PI: 0.2–0.75 Hz of the spectrum) estimates cardiac sympathetic activity. The high frequency band of the PI (HF-PI: 0,75–3 Hz of the spectrum) was used as an estimate of cardiac parasympathetic activity (Pagani et al., 1986; Kuwahara et al., 1994; De Andrade et al., 2014; Barros et al., 2015; Paulino-Silva and Costa-Silva, 2016). Sympathovagal balance was estimated by the LF/HF ratio in the PI spectrum (Barros et al., 2015; Paulino-Silva and Costa-Silva, 2016). Total variability of PI (Dobrek et al., 2013) was calculated from the sum of all the PI spectrum.

The spontaneous cardiac baroreflex sensitivity was evaluated through the sequence method using CardioSeries 2.4 Software (http://www.danielpenteado.com/, freely available), which calculates the spontaneous baroreflex through the angle of the linear regression between MAP and PI, with a delay of 3 heartbeats. Sequences with at least 3 intervals were considered only if the correlation of the coefficients was very high (>0.85). The spontaneous cardiac baroreflex sensitivity was estimated by the average of significant angles (Silva et al., 2015). The baroreflex effectiveness index (BEI) was calculated by the ratio between the total number of PI/MAP sequences and the total number of MAP ramps (Di Rienzo et al., 2001).

### 2.6 Sympathovagal Tone and Intrinsic Heart Rate

Baseline recordings of arterial pressure were performed on the first and second days of the experimental protocol. Then, methylatropine (3 mg/kg; i.v.; Sigma-Aldrich, San Luis, MO, EUA), a muscarinic antagonist, was used to estimate parasympathetic tone. After recovery, a selective β1 adrenergic antagonist, atenolol (4 mg/kg; i.v.; Sigma-Aldrich, San Luis, MO, EUA), was used for the assessment of cardiac sympathetic tone (Simas et al., 2018).

Heart rate was recorded for 15 min after injection of the first drug and, after stabilization, the second drug was infused to promote autonomic blockade. On the following day, the application sequence was performed in reversed order (Barros et al., 2015).

Analysis of the parasympathetic and sympathetic tone was achieved via the HR response to methylatropine and atenolol, respectively. Intrinsic HR was analyzed after each sequence of applications and averaged over the 2 days of the protocol (Barros et al., 2015; Simas et al., 2018).

### 2.7 Estimate of Induced Baroreflex Sensitivity

On the third day of the protocol, the induced baroreflex sensitivity was estimated by measuring responses to a vasopressor dose of the α1 adrenergic agonist phenylephrine (8 μg/kg; i. v.; Sigma-Aldrich, San Luis, MO, EUA). After a 20-minutes-recovery period a vasodepressor dose of intravenous sodium nitroprusside was delivered (50 μg/kg; i.v.; Sigma-Aldrich, San Luis, MO, EUA). The baroreflex sensitivity index was calculated from the HR variation as a function of MAP (ΔHR/ΔMAP) (Valenti et al., 2009; Valenti et al., 2011).

### 2.8 Vascular Sympathetic Tone

On the third day, 20 min after the evaluation of the induced baroreflex sensitivity and subsequent cardiovascular stabilization, an intravenous injection of a ganglion blocker, hexamethonium (30 mg/kg; i.v.; Sigma-Aldrich, San Luis, MO, EUA), was performed to assess the vascular sympathetic contribution to MAP (Radaelli et al., 2006). The vascular sympathetic tone was estimated by the MAP response to the ganglion blocker (Barros et al., 2015).

### 2.9 Oxidative Stress Parameters Assays

A separate cohort of animals was fasted for 12 hs then euthanized with guillotine (8:00–12:00) and the brain immediately removed. The brainstem was separated, snap-frozen and stored in liquid nitrogen. The thoracic cavity was opened, and the heart was removed, washed with 0.9% saline to remove blood, then snap frozen and stored in liquid nitrogen for storage at −80°C.

Frozen brainstem, and heart tissues were separately homogenized in van Potter-Elvehjem homogenizers with seven volumes of ice-cold 0.1 M potassium phosphate buffer (pH 7.4) and an aliquot was separated as total homogenate. The remaining homogenate was centrifuged (11,000 g/15 min) and the supernatant separated as soluble fractions of the homogenate.

#### 2.9.1 Protein Carbonyl Groups

The levels of protein carbonyl groups were measured spectrophotometrically using 2,4-dinitrophenylhydrazine (ε_370_ = 22 × 10^3^ M^−1^ cm^−1^) and the results were expressed as nmol (mg protein)^−1^ (Levine et al., 1990).

#### 2.9.2 Glutathione Assay

Reduced glutathione (GSH) and oxidized glutathione (GSSG) were measured in the total homogenate. GSH and GSSG contents were measured spectrofluorimetrically (excitation at 350 nm and emission at 420 nm) by means of o-phthalaldehyde (OPT) assay as previously described (Hissin and Hilf, 1976). The fluorescence was estimated as GSH. For GSSG assay, samples were pre-incubated with 10 mM N-ethylmaleimide for 20 min and then with a mixture containing 1 M NaOH and 0.4 µM OPT to detect the fluorescence. Standard curves were prepared with GSH or GSSG and the contents were expressed as nmol (mg protein)^−1^.

#### 2.9.3 Enzyme Activities

The activity of catalase (CAT) was estimated by measuring the change in absorbance at 240 nm using H_2_O_2_ as substrate and expressed as μmol min^−1^ (mg protein)^−1^ (Bergmeyer, 1974). Superoxide dismutase (SOD) activity was estimated by its capacity to inhibit pyrogallol autoxidation in an alkaline medium. The latter was measured at 420 nm (Marklund and Marklund, 1974). One SOD unit was considered the quantity of enzyme that was able to promote 50% inhibition and results are expressed as arbitrary units (mg protein)^−1^.

Protein content: total protein was assayed in the total homogenate and supernatant using the Folin phenol reagent (Lowry et al., 1951).

### 2.10 Histological Analysis of the Heart

After euthanasia with guillotine (8:00–12:00) the heart was immediately removed and frozen for histological and molecular analyses. Heart samples were fixed in formalin, dehydrated in graded alcohols and embedded in histological paraffin (Histopar, Easypath, São Paulo, Brazil). Blocks were sectioned on a microtome (RM2245, Leica Microsystems, Wetzlar, Germany) as 6 μm thick non-serial sections and stained with Picrosirius Red, for measurement of total collagen. Analysis of cardiomyocyte diameter was performed on Hematoxylin and Eosin stained sections.

Photomicrographs were taken on light microscope coupled to a digital camera (DM500 plus ICC50 HD, Leica Microsystems, Wetzlar, Germany), at x1000 for the measurement of the cardiomyocytes (100 cardiomyocytes/group), x100 for the measurement of perivascular fibrosis (25 images/group) and x100 for the measurement of interstitial fibrosis (50 images/group). Photomicrographs of cross sections of the left ventricular cardiomyocytes were analyzed. The distance between the upper and lower parts of the plasma membrane of each cardiomyocyte in a set field was measured.

The mean cardiomyocyte diameter was calculated for each animal. Photomicrographs containing collagen labeling in the fields with sectioned arterioles were analyzed. The perivascular fibrosis index (PFI) was determined by dividing the total area of fibrosis and the area of the vessel lumen.

Interstitial fibrosis was analyzed by stereology, using a mesh composed of 594 test points. The percentage of interstitial fibrosis was calculated by the proportion between the number of points that reach the red, marked collagen, and the total test points. The mean of both PFI and Interstitial Fibrosis for each animal were calculated. Cardiomyocyte diameter and PFI analyzes were performed using ICY software (Institut Pasteur, Paris, France, https://icy.bioimageanalysis.org/, freely available). Interstitial fibrosis was assessed using Image Pro Plus v6 (Media Cybernetics, MD, United States, https://www.mediacy.com/imageproplus, paid software).

### 2.11 Western Blotting in Heart

Left ventricle samples, collected after euthanasia with guillotine (8:00–12:00) and frozen for histological and molecular analyses, were homogenized in RIPA lysis buffer with a protease inhibitor cocktail. The supernatant total protein content was quantified by Bradford assay. Samples were denatured in Laemmli buffer at 95°C for 5 min then aliquots of 20 μg of proteins from each sample were subjected to separation by SDS-PAGE. Protein separation was accompanied by a positive control with pre-determined staining (EZ-Run, Fisher BioReagents, Göteborg, SE). Separated proteins on the gel were transferred to nitrocellulose membranes (Amersham Protran, GE Healthcare, Little Chalfont, BUX, United Kingdom) in a wet transfer system, soaked in transfer buffer then the membranes were incubated with a blocking buffer, and subsequently incubated with primary antibodies (Table 2). Membranes were gently washed and incubated with HRP-conjugated secondary antibody, and covered with chemiluminescence detection solution (Amersham ECL, GE Healthcare, Little Chalfont, BUX, United Kingdom). Chemiluminescence was detected by an image documentation system (ImageQuant LAS 600 series, GE Healthcare, Chicago, IL, United States). The intensity of the resultant bands was quantified by relative optical density using FIJI software (ImageJ, NIH, Cambridge, MA, United States, https://imagej.net/software/fiji/downloads, freely available). GAPDH (Glyceraldehyde-3-Phosphate Dehydrogenase) was used as load control.

**TABLE 2:** List of antibodies used for western immunoblotting.

Antibody	Manufacturer and Catalog	Source	Dilution
Anti-B1 AR	Thermo Fisher Scientific, MA United States (PA1-049)	Mouse monoclonal	1:1000
Anti α1 AR	Thermo Fisher Scientific, MA United States (PA1-047)	Mouse monoclonal	1:1000
Anti M1 R	Sigma Aldrich, MO United States (M9808)	Rabbit monoclonal	1:1000
Anti mTor	Thermo Fisher Scientific, MA United States	Mouse polyclonal	1:1000
Anti-SOD	Santa Cruz, CA, United States (SC-11407)	Rabbit polyclonal	1:1000
Anti-CAT	LifeSpan, WA, United States (LS-C21346)	Rabbit polyclonal	1:1000
Anti-GAPDH	Santa Cruz, CA, United States (SC-25778)	Rabbit polyclonal	1:1000

**TABLE 3:** Effects of dietary protein restriction in peri-pubertal period on biometric and biochemical parameters.

Biometric and biochemical parameters	NP	LP	*p* value
Body weight (g)	398 ± 589	359 ± 8.04	0.0003***
Body length (cm)	23.96 ± 0.09	22.95 ± 0.19	0.0002***
Mesenteric fat pad (g/100 g bw)	0.70 ± 0.03	0.67 ± 0.02	0.580
Retroperitoneal fat pad (g/100 g bw)	1.29 ± 0.05	1.27 ± 0.05	0.765
Blood glucose (mmol L^−1^)	89 ± 2.61	101 ± 3.11	0.008**
Triglycerides (μmol L^−1^)	126 ± 9.48	128 ± 4.59	0.859
Total cholesterol (μmol L^−1^)	89 ± 3.38	82 ± 1.55	0.087
HDL - cholesterol (μmol L-)	53 ± 1.64	57 ± 2.57	0.195

NP: Normal protein diet; LP: Low protein diet; n = 8–27 animals per group. Values expressed as mean ± SEM ***p < 0.001, **p < 0.01, and *p < 0.05 indicate statistical significance (Student’s T test).

### 2.12 Statistical Analysis

The data are presented as mean ± standard error of the mean (SEM). The statistical analysis was performed after analysis of data distribution (Shapiro Wilk test). Results are considered significant when *p* < 0.05. GraphPad Prism 6 (GraphPad software, La Jolla, CA, United States, https://www.graphpad.com/scientific-software/prism/, paid software) was used for graphical representation and statistical analysis and the comparison between the groups was performed using the Student’s T test or two-way ANOVA with time and diet as independent factors.

## 3 Results

### 3.1 Food Intake, Body Weight, and Growth Parameters

**FIGURE 1:**
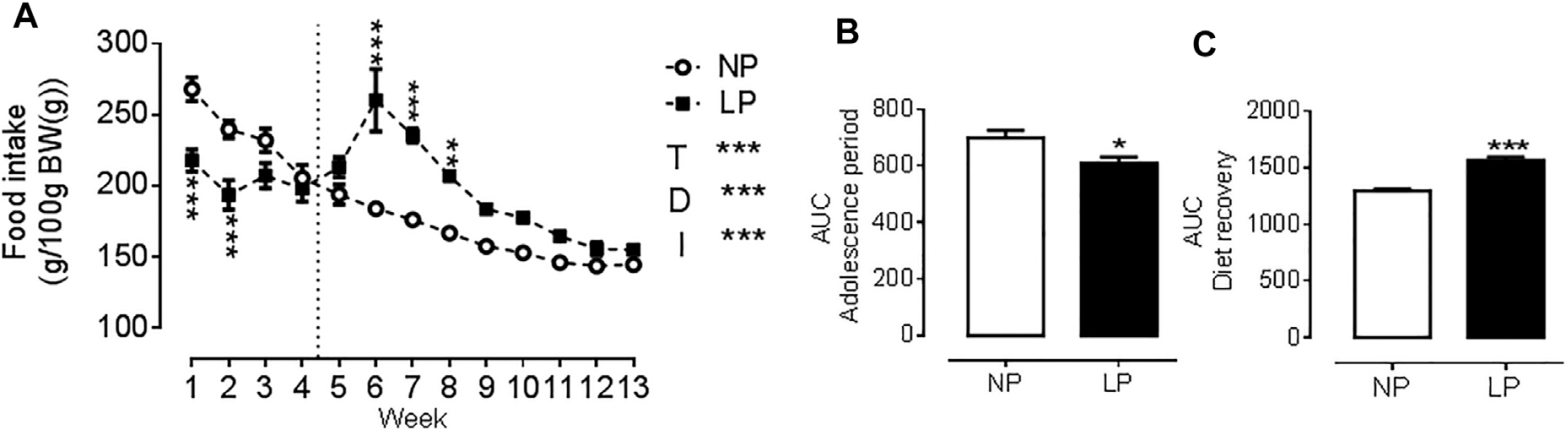
Food consumption throughout the experimental protocol. Food consumption **(A)**, Area under the curve of food consumption during the peri-pubertal period **(B)**, Area under the curve of food consumption during the dietary recovery period **(C)**, *n* = 18 animals per group. NP, normal protein diet; LP, low protein diet; I, interaction between diet and time, AUC, area under the curve. Values presented as mean ± SD. ***p < 0.001, **p < 0.01, and *p < 0.05 indicate statistical significance (Two-way ANOVA or Student’s T test).

Animals given a low protein diet during peri-puberty showed reduced food intake (LP: 608 ± 22.3 vs. NP: 699 ± 25 g/100 g BW; *p* = 0.021; Figure 1B.) over the peri-pubertal period, which also resulted in a reduction of total caloric intake of the same magnitude. In the period of dietary recovery, there was a rapid increase in food intake (LP: 1567 ± 28.7 vs. NP: 1295 ± 20.7 g/100 g BW; *p* = 0.001; Figure 1C.) but despite this catch-up growth LP animals had a lower body weight (LP: 359 ± 8.04 vs. NP: 398 ± 5.89 g; *p* = 0.003), with a reduction in naso-anal length (LP: 22.9 ± 0.19 vs. NP: 23.9 ± 0.09 cm; *p* = 0.0002) compared with controls at PN120 (Table 3).

LP animals showed an increase in fasting blood glucose (LP: 101.5 ± 3.11 vs. NP: 89.6 ± 2.61 mg/dl; *p* = 0.0081) compared with controls, but there was no change in the lipid profile. There was no significant difference in the mesenteric or retroperitoneal fat mass between groups (Table 3).

### 3.2 Arterial Pressure, Pulse Pressure, and Heart Rate

**FIGURE 2:**
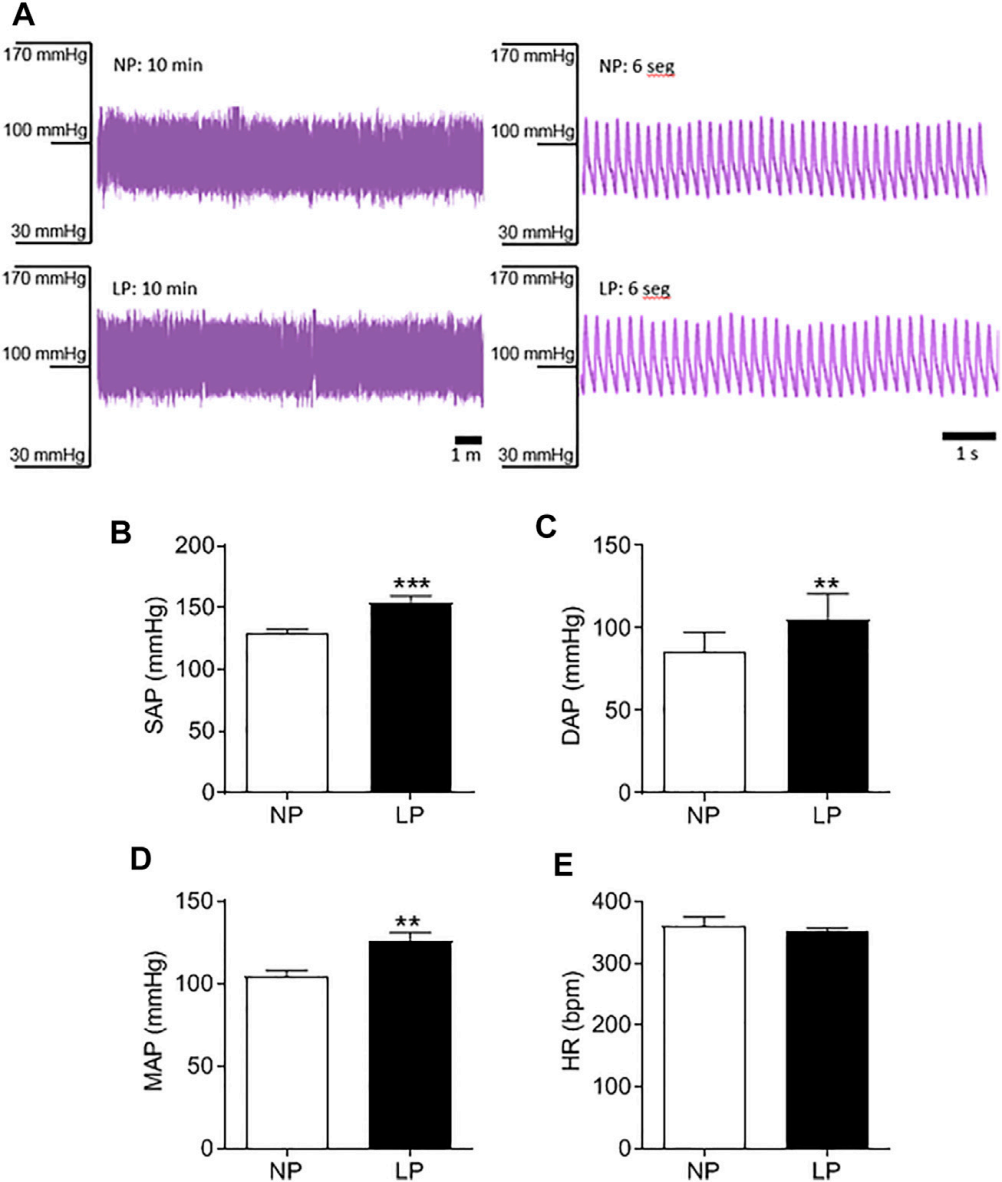
Basal arterial pressure in 120 days old animal. Representative blood pressure traces of NP and LP animals in basal conditions **(A)**, Systolic arterial pressure **(B)**, diastolic arterial pressure **(C)**, mean arterial pressure **(D)** and heart rate **(E)**, *n* = 9–10 animals per group. NP, normal protein diet; LP, low protein diet. Values presented as mean ± SD ***p < 0.001, **p < 0.01, and *p < 0.05 indicate statistical significance (Student’s T test).

Good quality blood pressure traces were achieved for all animals (representative data shown in Figure 2A). LP animals showed an increase in SAP (LP: 154 ± 4.84 vs. NP: 129 ± 3.08 mmHg; *p* = 0.0003; Figure 2B), DAP (LP: 105 ± 4.93 vs. NP: 85 ± 3.69 mmHg; *p* = 0.005; Figure 2C) and MAP (LP: 126 ± 4.93 vs. NP: 104 ± 3.37 mmHg; *p* = 0.002; Figure 2D). There was no significant difference in heart rate between groups (Figure 2E).

### 3.3 Cardiovascular Variability

**FIGURE 3:**
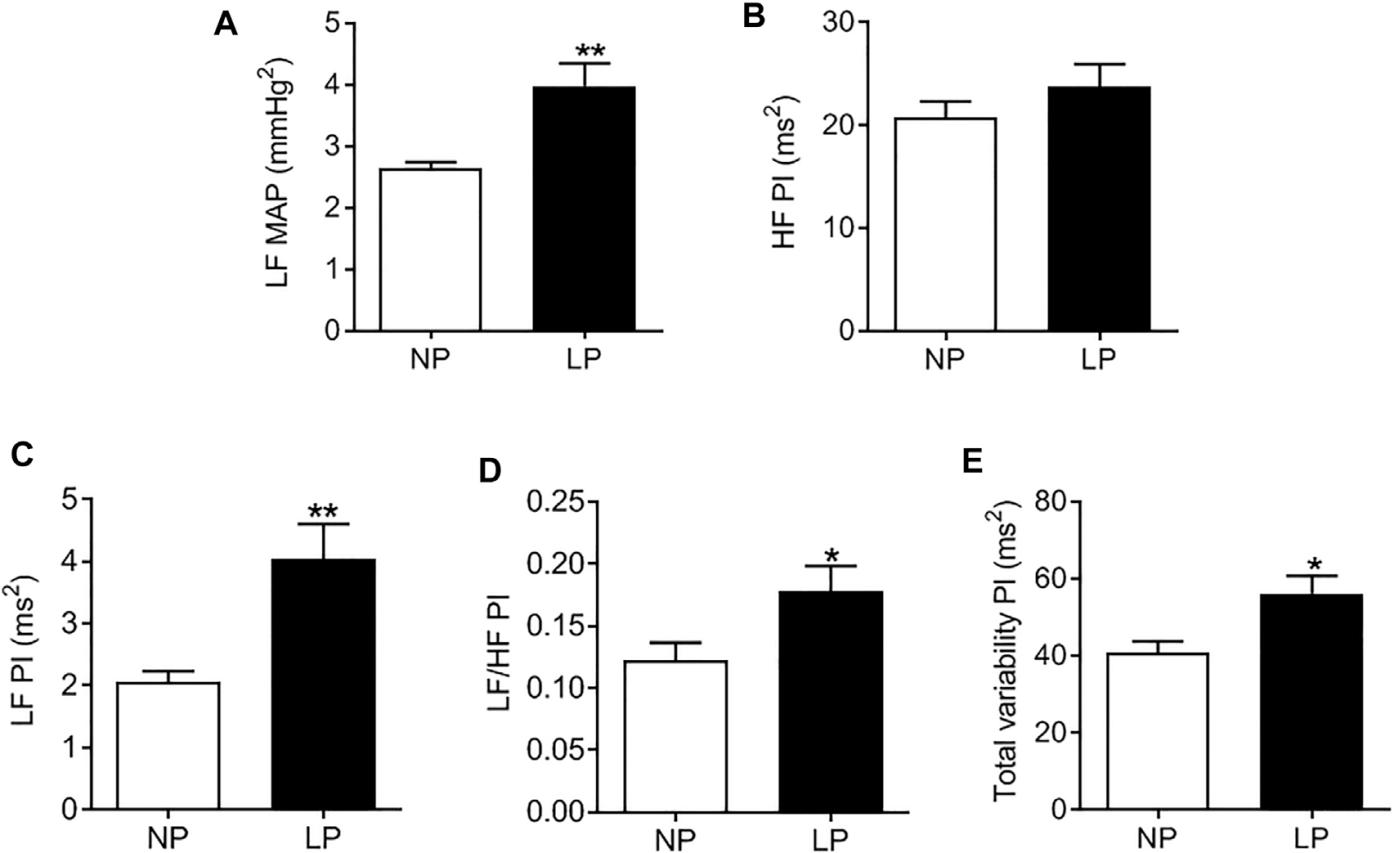
Spectral analysis of arterial pressure and pulse interval in 120-day-old animals at baseline conditions. Low frequency zone of mean arterial pressure **(A)**, high frequency zone of pulse interval **(B)**, low frequency zone of pulse interval **(C)**, low frequency/high frequency pulse interval **(D)** ratio, total variability of the pulse interval (E), *n* = 7–9 animals per group. NP, normal protein diet; LP, low protein diet. Values presented as mean ± SD ***p < 0.001, **p < 0.01, and *p < 0.05 indicate statistical significance (Student’s T test).

LP animals showed an increase in the LF-MAP band, indicating an increase in vascular sympathetic activity (LP: 3.9 ± 0.37 vs. NP: 2.8 ± 0.34 mmHg^2^; *p* = 0.080; Figure 3A). There was no change in the HF-PI band (Figure 3B). The LP group showed an increase in the LF-PI band (LP: 4.04 ± 0.56 vs. NP: 2.3 ± 0.28 ms^2^; *p* = 0.062; Figure 3C) and in the LF/HF ratio (LP: 0.17 ± 0.020 vs. NP: 0.12 ± 0.014; *p* = 0.048; Figure 3D) indicating an increase in sympathetic activity. The total PI variability (LP: 56 ± 4.49 vs. NP: 40 ± 3.03 ms^2^; *p* = 0.011; Figure 3E) was also increased in LP animals.

### 3.4 Sympathovagal Tone and Intrinsic Heart Rate

Cardiac vagal tone, evaluated with methylatropine, and cardiac sympathetic tone, evaluated with atenolol, were similar between groups. The intrinsic heart rate, assessed after double autonomic blockade with methylatropine and atenolol, was also not altered in the LP group compared with control animals (Table 4).

**TABLE 4:** Cardiovascular autonomic evaluation after drug injection.

Parameters	NP	LP	*p* value
Control of autonomic tone			
Δ HR after methylatropine, bpm	86.20 ± 4.61	82 ± 8.11	0.735
Δ HR after atenolol, bpm	−57 ± 7.73	–46 ± 4.22	0.208
Intrinsic heart rate, bpm	347 ± 7.52	357 ± 5.37	0.281
Baroreflex sensitivity			
BEI	0.20 ± 0.014	0.22 ± 0.018	0.391
GAIN (spontaneous baroreflex)	7.13 ± 0.79	6.21 ± 0.37	0.311
Bradycardic response, bpm/mmHg	–2.21 ± 0.40	–2.13 ± 0.25	0.802
Tachycardic response, bpm/mmHg	–3.03 ± 0.60	–3.40 ± 0.37	0.689
Δ SAP after phenylephrine, mmHg	60 ± 6.23	76 ± 4.14	0.049*
Δ SAP after sodium nitroprusside, mmHg	–45.83 ± 3.615	–45.37 ± 3.11	0.926

NP: Normal protein diet; LP: Low protein diet; Parasympathetic and sympathetic activity induced by methylatropine and atenolol (ΔHR, after application stabilization - basal HR); Intrinsic heart rate after double blocking with methylatropine and atenolol; Baroreflex effectiveness index (BEI); Gain baroreflex (GAIN); Baroreflex induced by phenylephrine and calculated sodium nitroprusside (ΔHR/ΔMAP); Pressure response to phenylephrine and sodium nitroprusside; n = 9–18 animals per group. Values exposed in mean ± SEM ***p < 0.001, **p < 0.01, and *p < 0.05 indicating statistical significance (Student T test).

### 3.5 Vascular Sympathetic Tone

**FIGURE 4:**
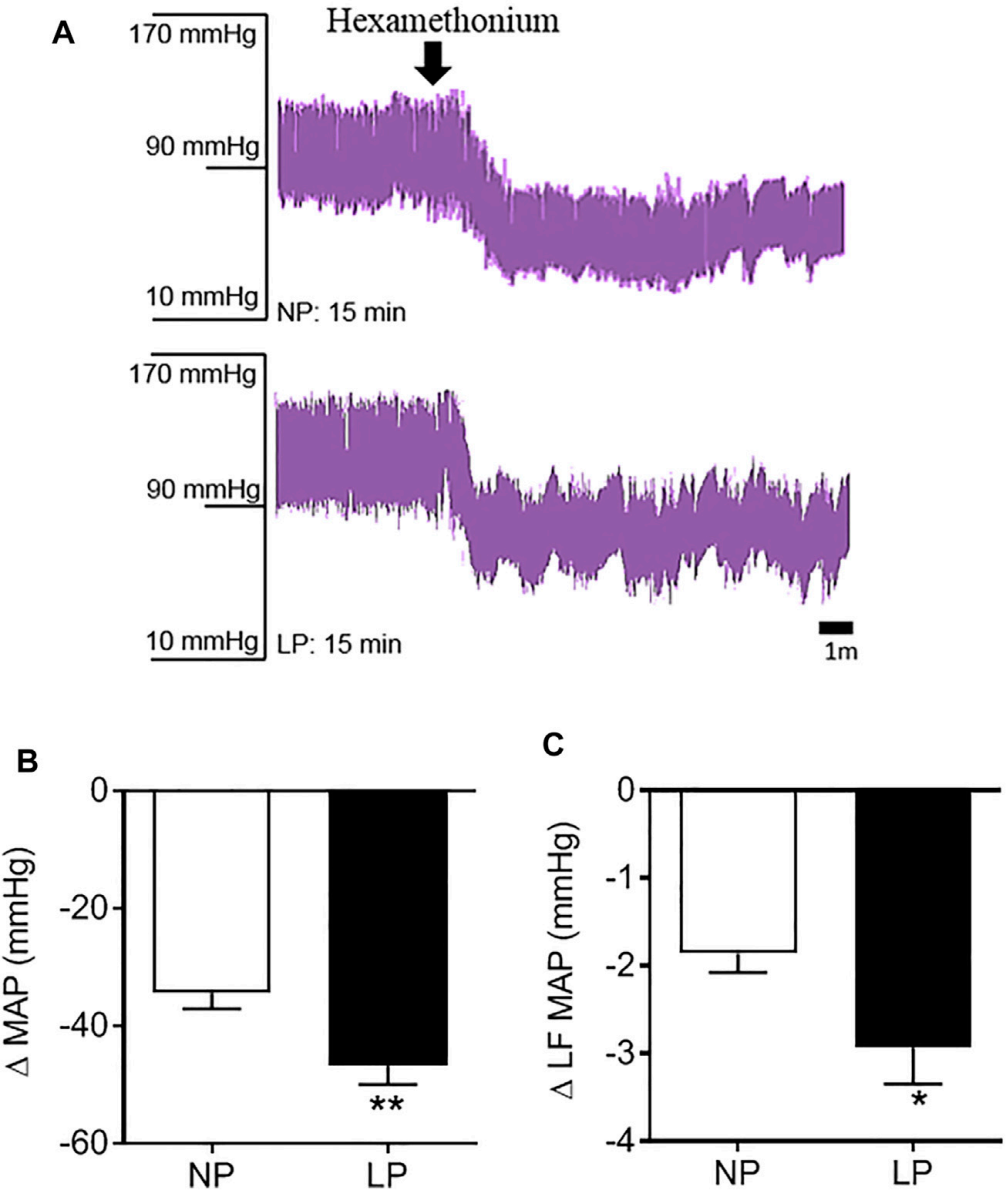
Pressure response to hexamethonium. Representative records of the depressor response to hexamethonium **(A)**, Change in mean arterial pressure **(B)**, and the low frequency zone of mean arterial pressure in response to hexamethonium **(C)**, *n* = 10–13 animals per group. NP, normal protein diet; LP, low protein diet. Values presented as mean ± SD ***p < 0.001, **p < 0.01, and *p < 0.05 indicate statistical significance (Student’s T test).

Representative records of the depressor response to hexamethonium, a ganglionic blocker, are shown in Figure 4A. LP animals showed a greater decrease in MAP (LP: −46 ± 3.11 vs. NP: −34 ± 2.63 mmHg; *p* = 0.006; Figure 4B) and amplitude of the LF-MAP zone after injection of hexamethonium (LP: −2.93 ± 0.41 vs. NP: −1.86 ± 0.21 mmHg^2^; *p* = 0.032; Figure 4C) compared with the control group. LP animals showed an increase in the pressure response to phenylephrine (LP: 76 ± 4.14 vs. NP: 60 ± 6.23 mmHg; *p* = 0.049), with no change in the pressure response to sodium nitroprusside (Table 4).

### 3.6 Sensitivity of Cardiac Baroreflex

The LP group showed no change in reflex bradycardia induced by phenylephrine or reflex tachycardia after sodium nitroprusside injection. The spontaneous baroreflex assessed by the BEI and gain, was similar between the groups (Table 4).

### 3.7 Oxidative Stress in the Brainstem

The levels of protein carbonyl groups were lower in LP animals compared with control rats (LP: 6 ± 0.52 vs. NP: 10 ± 0.55 mg/protein; *p* = 0.002). The activities of SOD (NP: 1.25 ± 0.03 vs. LP: 1.09 ± 0.06 U mg^−1^; *p* = 0.044) and CAT (LP: 23 ± 1.47 vs. NP: 30 ± 1.47 μmol min^−1^·mg^−1^; *p* = 0.012) were lower in the brainstem of rats fed a low protein diet (Table 5).

**TABLE 5:** Oxidative stress in brainstem.

Parameters	NP	LP	*p* value
Carbonyl protein groups	10.06 ± 0.55	6.69 ± 0.52	0.002**
GSH (nmol mg^−1^)	10.11 ± 0.62	12.91 ± 0.85	0.039*
GSSG (nmol mg^−1^)	0.15 ± 0.01	0.16 ± 0.01	>0.999
GSH + 2xGSSG (nmol mg^−1^)	10.41 ± 0.62	13.23 ± 0.85	0.038*
GSH/GSSG	67.4 ± 4.1	80.6 ± 5.3	0.101
SOD (U mg^−1^)	1.25 ± 0.03	1.09 ± 0.06	0.044*
Catalase (µmol·min^−1^ mg^−1^)	30.5 ± 1.5	23.8 ± 1.5	0.012*

NP: Normal protein diet; LP: Low protein diet; Carbonylated proteins, reduced glutathione (GSH), oxidized glutathione (GSSG) and activity of catalase (CAT) and superoxide dismutase enzymes (SOD); n = 4–7 animals per group. Values exposed in mean ± SEM ***p < 0.001, **p < 0.01, and *p < 0.05 indicating statistical significance (Student T test).

The levels of GSH (LP: 12 ± 0.85 vs. NP: 10 ± 0.62 nmol mg^−1^; *p* = 0.039) and total glutathione (LP: 13 ± 0.85 vs. NP: 10 ± 0.62 nmol mg^−1^; *p* = 0.038) were higher in the brainstem of LP rats. The levels of GSSG and the GSH/GSSG ratio were not different between the groups (Table 5).

### 3.8 Heart Histology

**FIGURE 5:**
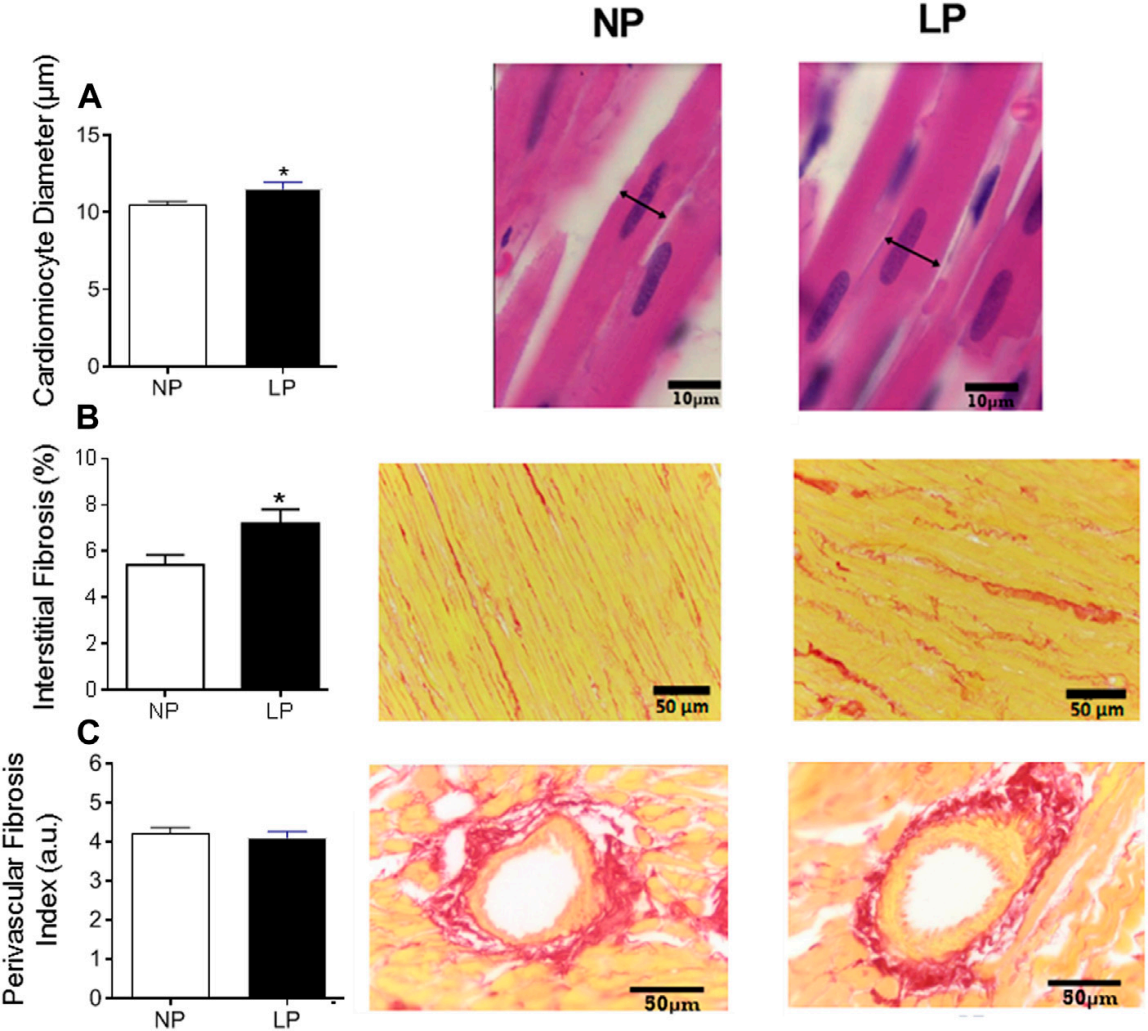
Histological analysis of the heart. Mean value of cardiomyocytes in cross section (scale bar = 10 μm) and representative pictures from each group **(A)**, cardiac interstitial fibrosis (scale bar = 50 μm) and representative pictures from each group **(B)** and perivascular fibrosis index in the left ventricular vasculature (scale bar = 50 μm) and representative pictures from each group **(C)**, *n* = 5–10 animals per group. NP, normal protein diet; LP, low protein diet. Values presented as mean ± SD ***p < 0.001, **p < 0.01, and *p < 0.05 indicate statistical significance (Student’s T test).

The LP group showed an increase in the cardiomyocyte diameter (LP: 11.50 ± 0.435 vs. NP: 10.44 ± 0.263 µm; *p* = 0.044; Figure 5A) and interstitial fibrosis (LP: 7.20 ± 0.59 vs. NP: 5.40 ± 0.43 µm; *p* = 0.028; Figure 5B), however, there was no change in perivascular fibrosis compared with the control group (Figure 5C).

### 3.9 Protein Abundance and Oxidative Stress in the Heart

The levels of protein carbonyl groups, a pro-oxidative marker, were lower in LP rats compared with NP animals (LP: 5 ± 0.56 vs. NP: 6 ± 0.33 mg/protein; *p* = 0.030). GSH and GSSG content in the heart were similar between the groups, however, CAT activity was lower in LP rats (LP: 19 ± 2.05 vs. NP: 32 ± 1.76 μmol min^−1^·mg^−1^; *p* = 0.001) (Table 6).

**TABLE 6:** Oxidative Stress and receptor protein abundance in Heart.

Parameters	NP	LP	*p* value
Oxidative stress			
Carbonyl protein groups	6.92 ± 0.33	5.01 ± 0.57	0.030*
GSH (nmol mg^−1^)	6.11 ± 0.33	5.11 ± 0.57	0.166
GSSG (nmol mg^−1^)	0.10 ± 0.01	0.10 ± 0.01	>0.999
GSH + 2xGSSG (nmol mg^−1^)	6.32 ± 0.33	5.31 ± 0.57	0.166
GSH/GSSG	60.9 ± 4.1	51.1 ± 5.7	0.200
Catalase (µmol·min^−1^ mg^−1^)	32.5 ± 1.7	19.9 ± 2.0	0.001**

NP, Normal protein diet; LP, Low protein diet; Carbonylated proteins, reduced glutathione (GSH), oxidized glutathione (GSSG), activity of catalase enzymes (CAT) and superoxide dismutase enzymes (SOD); *n* = 4–7 animals per group. Values exposed in mean ± SEM ***p < 0.001, **p < 0.01, and *p < 0.05 indicating statistical significance (Student T test).

**FIGURE 6:**
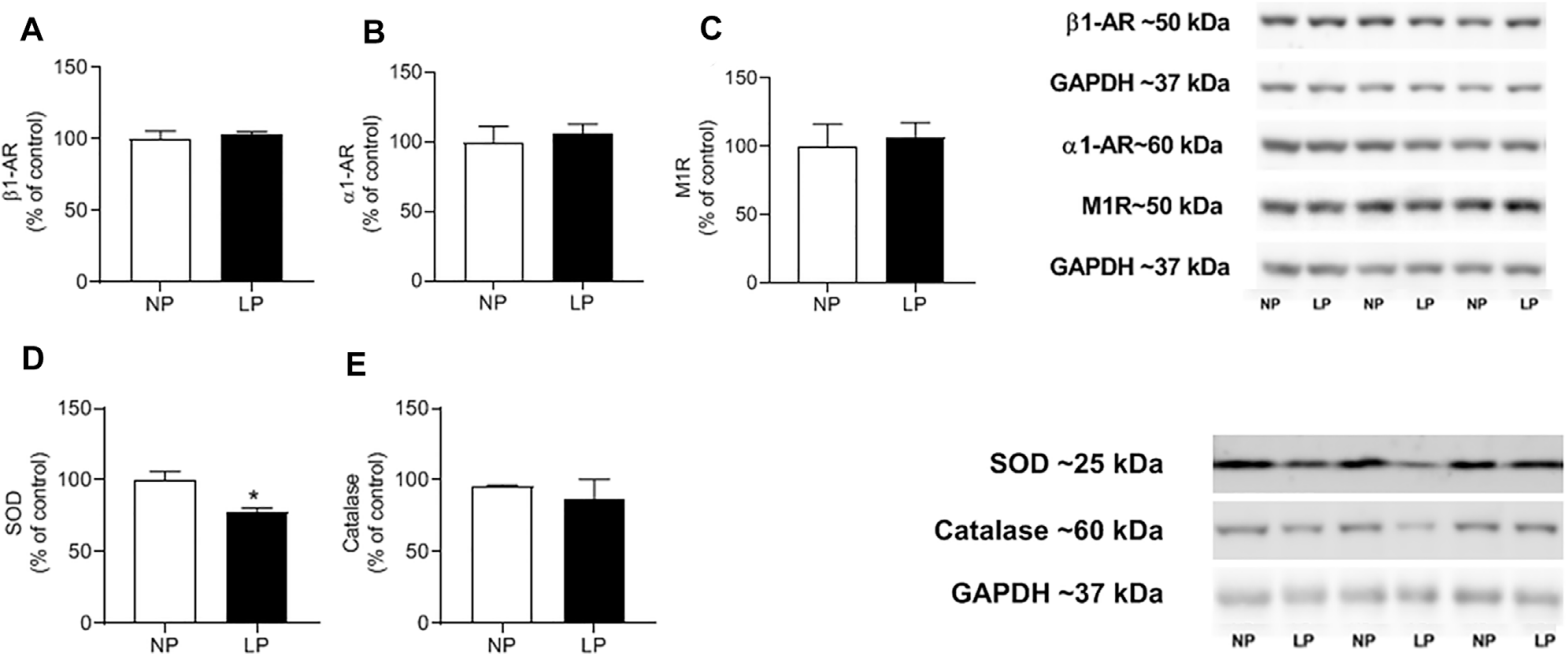
Cardiac protein expression. Mean value of western blot in beta-1 receptor (Anti-B1) **(A)**, alpha-1 receptor (Anti- α1) **(B)**, muscarinic-1 receptor (Anti- M1) **(C)** and superoxide dismutase enzymes (SOD) **(D)**, activity of catalase enzymes (CAT) **(E)** and representative immunoblots are show on the up-right corner, *n* = 4–7 animals per group. NP, normal protein diet; LP, low protein diet. Values presented as mean ± SD ***p < 0.001, **p < 0.01, and *p < 0.05 indicate statistical significance (Student’s T test).

In the heart, the abundance of beta 1, alpha 1, muscarinic 1, and catalase receptors were similar between groups. However, the expression of superoxide dismutase decreased in the LP group (LP: 77.85 ± 6.04 vs. 100 ± 2.78 kDa; *p* = 0.018) (Figure 6).

## 4 Discussion

This work demonstrates that dietary protein restriction in peri-pubertal period followed by 60 days of nutritional recovery results in hypertension in young adulthood which appears to be underpinned by sympathetic hyperactivity. This is the first demonstration of adult neurogenic hypertension programmed in the peri-pubertal period, while other studies have addressed the effect of low protein diet on blood pressure just after the exposure to the insult (Loss et al., 2007; Penitente et al., 2007; Del Carmen Miñana-Solis and Escobar, 2008; Martins et al., 2011; Murca et al., 2012; Gomide et al., 2013; Penitente et al., 2014; Cappelli et al., 2018). In the context of DOHaD concept the studies have explored an earlier susceptible window of development, the peri-natal period (Latorraca et al., 1998; De Oliveira et al., 2011; De Brito Alves et al., 2014; De Oliveira et al., 2014; Nascimento et al., 2014; Barros et al., 2015; De Oliveira et al., 2016; Ferreira et al., 2016; Paulino-Silva and Costa-Silva, 2016; Rossini et al., 2017; Barros et al., 2018; Martins et al., 2018; Assalin et al., 2019; Ferreira et al., 2019). The exact stimulus to the neurogenic hypertension is difficult to determine as the LP animals were hypophagic during the period of protein restriction, which also induced a caloric-restriction in peri-puberty, and a critical window of development (De Oliveira et al., 2013; De Oliveira et al., 2018). When reverted to the control diet, LP animals were hyperphagic, and therefore demonstrated a catch-up growth, albeit without evidence of elevated adipose tissue accumulation, which is also known in peri-natal programmed hypertension. In young adult life, at PN120, LP animals showed an increase in arterial pressure, cardiac (LF-PI) and vascular sympathetic tone (LF-MAP), an exacerbated depressor response to hexamethonium and greater reduction in the LF-MAP band in response to hexamethonium, indicating sympathetic arousal. In addition, LP animals demonstrated cardiac remodeling in the form of cardiomyocyte hypertrophy and increased interstitial fibrosis, without elevated perivascular collagen deposition. These alterations point to a precocious cardiac remodeling process, which may be a consequence of the hypertension or altered sympathovagal tone. These changes are associated with disorganization of the redox state in the heart and brainstem stimulated by early exposure to malnutrition, which may be responsible for the altered autonomic balance.

Rat models of developmentally programmed hypertension following perinatal protein restriction (De Brito Alves et al., 2014; Barros et al., 2015; Paulino-Silva and Costa-Silva, 2016) suggest an increase in arterial pressure, cardiac sympathetic tonus and a vascular sympathetic hyperactivity, suggesting that protein restriction at critical stages of development contributes to increased sympathetic activity (Martins et al., 2011; Barros et al., 2015; Paulino-Silva and Costa-Silva, 2016). The present study shows that LP exposure in the peri-pubertal period (a later susceptible window of development) induces an increase in systolic, diastolic, and mean arterial pressure and cardiovascular sympathetic hyperactivity by young adulthood. These changes may be dependent on precocious changes to cardiac structure induced by the early insult. Previous studies point to an increase in blood pressure and sympathetic activity immediately after the caloric-protein insult during post-weaning/peri-pubertal period (Loss et al., 2007; Penitente et al., 2007; Murca et al., 2012; Penitente et al., 2014). We suggest that the autonomic dysfunction and hypertension observed in the present adult LP animals, exposed to protein restriction in peri-puberty, may be an indicative of hypertension developed in the peri-pubertal period that was maintained until adulthood.

During the exposure to the low protein diet, food intake was reduced, which leads to a caloric protein restriction, as observed previously (De Oliveira et al., 2013; De Oliveira et al., 2018). This pattern of ingestion may add confounding factors in the mechanism of blood pressure regulation as caloric restriction may also reduce blood pressure, secondary weight loss, and lowering of insulin resistance (Nicoll and Henein, 2018). Paradoxically, the benefit of a low protein diet on metabolism and blood pressure is controversial and may depend on the developmental window of the exposure and type of macronutrients that are substituted for protein (Laeger et al., 2018). Considering the previously described insulin resistance in this animal model of programming (De Oliveira et al., 2013) and the present increase in blood pressure we may suggest that here, the deleterious effect of low protein diet outweighs any putative beneficial effects of the caloric restriction. Furthermore, it is important to consider that in the present study animals are exposed to a dietary recovery of 60 days between the dietary insult and the timepoint of parameters’ evaluation. Interestingly, the changes observed in response to protein restriction followed by catchup growth mirror those of other models where animals exposed to an obesogenic diet in early life or in adulthood also develop elevated blood pressure driven by sympathetic activation, cardiac remodeling and biochemical as well as molecular biological changes in the brain (Prior et al., 2014; Lim et al., 2016; Barrand et al., 2017; De Jong et al., 2018). It is yet to be determined whether the primary driver is adiposity or the protein restriction *per se*, however the similarity in phenotype between these disparate models may indicate that modification in adipocyte biology or activity may be a contributor to the overall mechanism.

The sympathetic hyperactivity observed in LP animals may be related to increased vasoconstrictor response dependent on the activity of the alpha-1 adrenergic receptor, given the increased pressure response to phenylephrine. However, there is also strong evidence for a neurogenic basis for the hypertension, as the LP animals showed exacerbated hypotension and a greater fall in the variability of the LF-AP band when exposed to hexamethonium, a ganglionic blocker. A similar profile was observed in offspring animals exposed to maternal low protein diet (Barros et al., 2015). Furthermore, other models of hypertension as the SHR animals, a model widely known for its neurogenic hypertension (Li et al., 2015) and in obesity related hypertension, which also has a neurogenic origin (Prior et al., 2010; Armitage et al., 2012; Burke et al., 2013) also exhibited a greater drop in blood pressure and low-frequency band of blood pressure in response hexamethonium infusion.

The sensitivity of spontaneous or induced baroreflex was not altered in the present study, corroborating with other programed hypertension models induced by perinatal LP diet exposure (Barros et al., 2015; Paulino-Silva and Costa-Silva, 2016). The absence of altered baroreflex sensitivity may be a consequence of baroreflex resetting (Loss et al., 2007; Penitente et al., 2007; Murca et al., 2012), due to chronic elevation of arterial pressure, however, this aspect was not explored in the present study.

Models of hypertension programmed by perinatal LP diet exposure are associated with evidence of increased sympathetic activity and oxidative stress and decrease in antioxidant defenses in the brainstem (Ferreira et al., 2016; Ferreira et al., 2019) and in the heart (Nascimento et al., 2014). The present study shows a disorganization of these antioxidant pathways rather than a wholesale shift towards oxidative stress. We observed a decrease in the product of protein oxidation in the heart and brainstem. A similar profile was observed by Ferreira et al. (2019), who showed that carbonylated proteins and SOD were decreased, while malonildialdehyde, a lipid peroxidation marker, and glutathione transferase enzyme were increased in the brainstem in PN150 rats exposed to perinatal protein restriction (Ferreira et al., 2019). Oxidative stress is a mechanism involved in the sympathetic arousal generated from the brainstem and resulting in cardiac remodeling (Rossini et al., 2017; Assalin et al., 2019). Therefore, the present disorganization of the redox state must be better explored to assess its role as a mediator of the observed dysfunctions.

The increase in the cardiomyocyte diameter and interstitial fibrosis in relatively young animals exposed to the peri-pubertal protein restriction, and in the absence of perivascular fibrosis observed, suggests that we are observing the beginning of a cardiac remodeling process following elevated blood pressure and ANS dysregulation. Programming studies with LP diet during pregnancy point to a more advanced cardiac morphological disorganization at PN60 or PN112, including collagen deposition in the heart (Rossini et al., 2017; Assalin et al., 2019), decreased number of cardiomyocytes (Silva et al., 2013; Rossini et al., 2017; Assalin et al., 2019), and changes in heart expression of miRNA related to cardiac structure and function (Assalin et al., 2019). The difference in the cardiac histological profile observed between perinatal insult versus the insult during peri-puberty may indicate that peri-pubertal period is a less vulnerable period in terms of disease acquisition, or it may simply represent a rightward shift in the curve. Future studies are required to better understand the natural history of this programmed cardiovascular disease, among them, we emphasize the importance of recording blood pressure from peri-pubertal period until adulthood using telemetric device, to explore the time course of the blood pressure increase and the evolution of the autonomic dysfunction observed at PN120.

The present study showed that caloric protein restriction during peri-pubertal period induces a neurogenic form of hypertension in adulthood, underpinned by greater sympathetic activity and associated with a disorganization of the cerebral redox state, and structural cardiac alterations. These outcomes, if transferrable to humans, further highlights the relevance of a balanced diet in peri-pubertal period for the prevention and control of the hypertension pandemic. This study points to pathophysiological mechanisms of hypertension and its close relationship with populations in underdeveloped and developing countries exposed to protein and caloric restriction. Taken together, these results are relevant to reflections on public policies for the control of cardiovascular risk, especially in populations vulnerable to transient malnutrition.

## Data Availability

The raw data supporting the conclusion of this article will be made available by the authors, without undue reservation.

## References

[B1] Armitage J. A. Burke S. L. Prior L. J. Barzel B. Eikelis N. Lim K. (2012). Rapid Onset of Renal Sympathetic Nerve Activation in Rabbits Fed a High-Fat Diet. Hypertension 60, 163–171. 10.1161/hypertensionaha.111.190413 22647890

[B2] Assalin H. B. Gontijo J. A. R. Boer P. A. (2019). miRNAs, Target Genes Expression and Morphological Analysis on the Heart in Gestational Protein-Restricted Offspring. PLoS One 14, e0210454. 10.1371/journal.pone.0210454 31034522 PMC6507319

[B3] Barker D. Eriksson J. Forsén T. Osmond C. (2002). Fetal Origins of Adult Disease: Strength of Effects and Biological Basis. Int. J. Epidemiol. 31, 1235–1239. 10.1093/ije/31.6.1235 12540728

[B4] Barker D. J. (2004). The Developmental Origins of Chronic Adult Disease. Acta Paediatr. Suppl. 93, 26–33. 10.1111/j.1651-2227.2004.tb00236.x 15702667

[B5] Barrand S. Crowley T. M. Wood-Bradley R. J. De Jong K. A. Armitage J. A. (2017). Impact of Maternal High Fat Diet on Hypothalamic Transcriptome in Neonatal Sprague Dawley Rats. PLoS ONE 12, e0189492. 10.1371/journal.pone.0189492 29240779 PMC5730210

[B6] Barros M. A. V. De Brito Alves J. L. Nogueira V. O. Wanderley A. G. Costa-Silva J. H. (2015). Maternal Low-Protein Diet Induces Changes in the Cardiovascular Autonomic Modulation in Male Rat Offspring. Nutr. Metab. Cardiovasc. Dis. 25, 123–130. 10.1016/j.numecd.2014.07.011 25287449

[B7] Barros M. A. V. Andrade E. B. Barros R. G. N. Costa I. K. M. Costa I. C. L. Vitorino G. F. A. (2018). Low-protein Diet Does Not Alter Reproductive, Biochemical, and Hematological Parameters in Pregnant Wistar Rats. Braz. J. Med. Biol. Res. 51, e6602. 10.1590/1414-431x20186602 29791594 PMC6002141

[B8] Bergmeyer H. U. (1974). Methods of Enzymatic Analysis. Weinheim: Chemie-Academic Press.

[B9] Burke S. L. Prior L. J. Lukoshkova E. V. Lim K. Barzel B. Davern P. J. (2013). Reduced Preprandial Dipping Accounts for Rapid Elevation of Blood Pressure and Renal Sympathetic Nerve Activity in Rabbits Fed a High-Fat Diet. Chronobiol. Int. 30, 726–738. 10.3109/07420528.2013.784771 23688116

[B10] Cappelli A. P. G. Zoppi C. C. Silveira L. R. Batista T. M. Paula F. M. da Silva P. M. R. (2018). Reduced Glucose‐induced Insulin Secretion in Low‐protein‐fed Rats Is Associated with Altered Pancreatic Islets Redox Status. J. Cel Physiol. 233, 486–496. 10.1002/jcp.25908 28370189

[B11] De Andrade O. Borghi S. M. De Souza H. C. D. Fontes M. A. P. Martins-Pinge M. C. (2014). Paraventricular Nucleus of Hypothalamus Participates in the Sympathetic Modulation and Spontaneous Fluctuation of Baroreflex during Head up Tilt in Unanesthetized Rats. Neurosci. Lett. 558, 1–7. 10.1016/j.neulet.2013.09.039 24176880

[B12] De Brito Alves J. L. Nogueira V. O. De Oliveira G. B. Da Silva G. S. F. Wanderley A. G. Leandro C. G. (2014). Short- and Long-Term Effects of a Maternal Low-Protein Diet on Ventilation, O2/CO2chemoreception and Arterial Blood Pressure in Male Rat Offspring. Br. J. Nutr. 111, 606–615. 10.1017/S0007114513002833 24059468

[B13] De Jong K. A. Barrand S. Wood-Bradley R. J. De Almeida D. L. Czeczor J. K. Lopaschuk G. D. (2018). Maternal High Fat Diet Induces Early Cardiac Hypertrophy and Alters Cardiac Metabolism in Sprague Dawley Rat Offspring. Nutr. Metab. Cardiovasc. Dis. 28, 600–609. 10.1016/j.numecd.2018.02.019 29691147

[B14] De Oliveira J. C. Scomparin D. X. Andreazzi A. E. Branco R. C. S. Martins A. G. Gravena C. (2011). Metabolic Imprinting by Maternal Protein Malnourishment Impairs Vagal Activity in Adult Rats. J. Neuroendocrinol. 23, 148–157. 10.1111/j.1365-2826.2010.02095.x 21091554

[B15] De Oliveira J. C. Lisboa P. C. De Moura E. G. Barella L. F. Miranda R. A. Malta A. (2013). Poor Pubertal Protein Nutrition Disturbs Glucose-Induced Insulin Secretion Process in Pancreatic Islets and Programs Rats in Adulthood to Increase Fat Accumulation. J. Endocrinol. 216, 195–206. 10.1530/JOE-12-0408 23151360

[B16] De Oliveira J. C. Miranda R. A. Barella L. F. Torrezan R. Agostinho A. R. Ribeiro T. A. S. (2014). Impaired β-cell Function in the Adult Offspring of Rats Fed a Protein-Restricted Diet during Lactation Is Associated with Changes in Muscarinic Acetylcholine Receptor Subtypes. Br. J. Nutr. 111, 227–235. 10.1017/s0007114513002213 23841989

[B17] De Oliveira J. C. Gomes R. M. Miranda R. A. Barella L. F. Malta A. Martins I. P. (2016). Protein Restriction during the Last Third of Pregnancy Malprograms the Neuroendocrine Axes to Induce Metabolic Syndrome in Adult Male Rat Offspring. Endocrinology 157, 1799–1812. 10.1210/en.2015-1883 27007071 PMC5393358

[B18] De Oliveira J. C. De Moura E. G. Miranda R. A. De Moraes A. M. P. Barella L. F. da Conceição E. P. S. (2018). Low-protein Diet in Puberty Impairs Testosterone Output and Energy Metabolism in Male Rats. J. Endocrinol. 237, 243–254. 10.1530/JOE-17-0606 29599416

[B19] del Carmen Miñana-Solis M. Escobar C. (2008). Post-weaning Protein Malnutrition in the Rat Produces Short and Long Term Metabolic Impairment, in Contrast to Earlier and Later Periods. Int. J. Biol. Sci. 4, 422–432. 10.7150/ijbs.4.422 19043606 PMC2586678

[B20] Di Rienzo M. Parati G. Castiglioni P. Tordi R. Mancia G. Pedotti A. (2001). Baroreflex Effectiveness index: an Additional Measure of Baroreflex Control of Heart Rate in Daily Life. Am. J. Physiol. Regulatory Integr. Comp. Physiol. 280, R744–R751. 10.1152/ajpregu.2001.280.3.r744 11171653

[B21] Dobrek L. Skowron B. Baranowska A. Thor P. J. (2013). Spectral Heart Rate Variability Analysis in Experimental Obstructive and Chemical Overactive Bladder Models. Adv. Clin. Exp. Med. 22, 337–346. 23828674

[B22] Ferreira D. S. Liu Y. Fernandes M. P. Lagranha C. J. (2016). Perinatal Low-Protein Diet Alters Brainstem Antioxidant Metabolism in Adult Offspring. Nutr. Neurosci. 19, 369–375. 10.1179/1476830515Y.0000000030 26035485

[B23] Ferreira D. J. S. Pedroza A. A. Braz G. R. F. Fernandes M. P. Lagranha C. J. (2019). Mitochondrial Dysfunction: Maternal Protein Restriction as a Trigger of Reactive Species Overproduction and Brainstem Energy Failure in Male Offspring Brainstem. Nutr. Neurosci. 22, 778–788. 10.1080/1028415x.2018.1444543 29495951

[B24] Gomide J. M. C. De Menezes R. C. Fernandes L. G. Silva F. C. Cardoso L. M. Miranda P. H. (2013). Increased Activity of the Renin-Angiotensin and Sympathetic Nervous Systems Is Required for Regulation of the Blood Pressure in Rats Fed a Low-Protein Diet. Exp. Physiol. 98, 57–66. 10.1113/expphysiol.2012.066712 22730415

[B25] Hissin P. J. Hilf R. (1976). A Fluorometric Method for Determination of Oxidized and Reduced Glutathione in Tissues. Anal. Biochem. 74, 214–226. 10.1016/0003-2697(76)90326-2 962076

[B26] Kuwahara M. Yayou K.-i. Ishii K. Hashimoto S.-i. Tsubone H. Sugano S. (1994). Power Spectral Analysis of Heart Rate Variability as a New Method for Assessing Autonomic Activity in the Rat. J. Electrocardiol. 27, 333–337. 10.1016/s0022-0736(05)80272-9 7815012

[B27] Laeger T. Castaño-Martinez T. Werno M. W. Japtok L. Baumeier C. Jonas W. (2018). Dietary Carbohydrates Impair the Protective Effect of Protein Restriction against Diabetes in NZO Mice Used as a Model of Type 2 Diabetes. Diabetologia 61, 1459–1469. 10.1007/s00125-018-4595-1 29550873 PMC6449005

[B28] Latorraca M. Q. Reis M. A. B. Carneiro E. M. Mello M. A. R. Velloso L. A. Saad M. J. A. (1998). Protein Deficiency and Nutritional Recovery Modulate Insulin Secretion and the Early Steps of Insulin Action in Rats. J. Nutr. 128, 1643–1649. 10.1093/jn/128.10.1643 9772130

[B29] Levine R. L. Garland D. Oliver C. N. Amici A. Climent I. Lenz A.-G. (1990). [49] Determination of Carbonyl Content in Oxidatively Modified Proteins. Methods Enzymol. 186, 464–478. 10.1016/0076-6879(90)86141-h 1978225

[B30] Li P. Gong J.-X. Sun W. Zhou B. Kong X.-Q. (2015). Hexamethonium Attenuates Sympathetic Activity and Blood Pressure in Spontaneously Hypertensive Rats. Mol. Med. Rep. 12, 7116–7122. 10.3892/mmr.2015.4315 26397056

[B31] Lim K. Barzel B. Burke S. L. Armitage J. A. Head G. A. (2016). Origin of Aberrant Blood Pressure and Sympathetic Regulation in Diet-Induced Obesity. Hypertension 68, 491–500. 10.1161/hypertensionaha.116.07461 27296999

[B32] Lim K. Burke S. L. Marques F. Z. Jackson K. L. Gueguen C. Sata Y. (2021). Leptin and Melanocortin Signaling Mediates Hypertension in Offspring from Female Rabbits Fed a High-Fat Diet during Gestation and Lactation. Front. Physiol. 12, 890. 10.3389/fphys.2021.693157 PMC8264761 34248679

[B33] Loss I. d. O. Fernandes L. G. Martins C. D. D. Cardoso L. M. Silva M. E. Dias-da-Silva V. J. (2007). Baroreflex Dysfunction in Rats Submitted to Protein Restriction. Life Sci. 81, 944–950. 10.1016/j.lfs.2007.08.005 17822720

[B34] Lowry O. Rosebrough N. Farr A. L. Randall R. (1951). Protein Measurement with the Folin Phenol Reagent. J. Biol. Chem. 193, 265–275. 10.1016/s0021-9258(19)52451-6 14907713

[B35] Marklund S. Marklund G. (1974). Involvement of the Superoxide Anion Radical in the Autoxidation of Pyrogallol and a Convenient Assay for Superoxide Dismutase. Eur. J. Biochem. 47, 469–474. 10.1111/j.1432-1033.1974.tb03714.x 4215654

[B36] Martins C. D. Chianca D. A. Jr. Fernandes L. G. (2011). Cardiac Autonomic Balance in Rats Submitted to Protein Restriction after Weaning. Clin. Exp. Pharmacol. Physiol. 38, 89–93. 10.1111/j.1440-1681.2010.05468.x 21143492

[B37] Martins I. P. De Oliveira J. C. Pavanello A. Matiusso C. C. I. Previate C. Tófolo L. P. (2018). Protein-restriction Diet during the Suckling Phase Programs Rat Metabolism against Obesity and Insulin Resistance Exacerbation Induced by a High-Fat Diet in Adulthood. J. Nutr. Biochem. 57, 153–161. 10.1016/j.jnutbio.2018.03.017 29730509

[B38] Mathers C. D. Loncar D. (2006). Projections of Global Mortality and Burden of Disease from 2002 to 2030. Plos Med. 3, e442. 10.1371/journal.pmed.0030442 17132052 PMC1664601

[B39] Mizuno M. Siddique K. Baum M. Smith S. A. (2013). Prenatal Programming of Hypertension Induces Sympathetic Overactivity in Response to Physical Stress. Hypertension 61, 180–186. 10.1161/hypertensionaha.112.199356 23150514 PMC3525329

[B40] Murca T. M. Magno T. S. d. R. De Maria M. L. d. A. Capuruço C. A. B. Chianca D. A. Ferreira A. J. (2012). Cardiac Responses of Rats Submitted to Postnatal Protein Restriction. Appl. Physiol. Nutr. Metab. 37, 455–462. 10.1139/h2012-017 22497279

[B41] Nascimento L. Freitas C. M. Silva-Filho R. Leite A. C. R. Silva A. B. da Silva A. I. (2014). The Effect of Maternal Low-Protein Diet on the Heart of Adult Offspring: Role of Mitochondria and Oxidative Stress. Appl. Physiol. Nutr. Metab. 39, 880–887. 10.1139/apnm-2013-0452 24905448

[B42] Nicoll R. Henein M. (2018). Caloric Restriction and its Effect on Blood Pressure, Heart Rate Variability and Arterial Stiffness and Dilatation: a Review of the Evidence. Int. J. Mol. Sci. 19, 751. 10.3390/ijms19030751 PMC5877612 29518898

[B43] Pagani M. Lombardi F. Guzzetti S. Rimoldi O. Furlan R. Pizzinelli P. (1986). Power Spectral Analysis of Heart Rate and Arterial Pressure Variabilities as a Marker of Sympatho-Vagal Interaction in Man and Conscious Dog. Circ. Res. 59, 178–193. 10.1161/01.res.59.2.178 2874900

[B44] Paulino-Silva K. M. Costa-Silva J. H. (2016). Hypertension in Rat Offspring Subjected to Perinatal Protein Malnutrition Is Not Related to the Baroreflex Dysfunction. Clin. Exp. Pharmacol. Physiol. 43, 1046–1053. 10.1111/1440-1681.12628 27463388

[B45] Penitente A. R. Fernandes L. G. Cardoso L. M. Silva M. E. Pedrosa M. L. Silva A. L. (2007). Malnutrition Enhances Cardiovascular Responses to Chemoreflex Activation in Awake Rats. Life Sci. 81, 609–614. 10.1016/j.lfs.2007.07.006 17688888

[B46] Penitente A. R. Novaes R. D. Silva M. E. Silva M. F. Quintão-Júnior J. F. Guatimosim S. (2014). Basal and β-Adrenergic Cardiomyocytes Contractility Dysfunction Induced by Dietary Protein Restriction Is Associated with Downregulation of SERCA2a Expression and Disturbance of Endoplasmic Reticulum Ca2+Regulation in Rats. Cell Physiol. Biochem. 34, 443–454. 10.1159/000363013 25095801

[B47] Prior L. J. Eikelis N. Armitage J. A. Davern P. J. Burke S. L. Montani J.-P. (2010). Exposure to a High-Fat Diet Alters Leptin Sensitivity and Elevates Renal Sympathetic Nerve Activity and Arterial Pressure in Rabbits. Hypertension 55, 862–868. 10.1161/hypertensionaha.109.141119 20194306

[B48] Prior L. J. Davern P. J. Burke S. L. Lim K. Armitage J. A. Head G. A. (2014). Exposure to a High-Fat Diet during Development Alters Leptin and Ghrelin Sensitivity and Elevates Renal Sympathetic Nerve Activity and Arterial Pressure in Rabbits. Hypertension 63, 338–345. 10.1161/hypertensionaha.113.02498 24191287

[B49] Radaelli A. Castiglioni P. Centola M. Cesana F. Balestri G. Ferrari A. U. (2006). Adrenergic Origin of Very Low-Frequency Blood Pressure Oscillations in the Unanesthetized Rat. Am. J. Physiol. Heart Circulatory Physiol. 290, H357–H364. 10.1152/ajpheart.00773.2005 16143647

[B50] Rodrigues F. A. Chianca-Jr D. A. Jr. Gonçalves Fernandes L. (2012). Malnutrition Affects the Pressor Response to Microinjection of L-Glutamate into the RVLM of Awake Rats. Biol. Res. 45, 337–343. 10.4067/S0716-97602012000400002 23558988

[B51] Rossini K. F. Oliveira C. A. d. Rebelato H. J. Esquisatto M. A. M. Catisti R. (2017). Gestational Protein Restriction Increases Cardiac Connexin 43 mRNA Levels in Male Adult Rat Offspring. Arq Bras Cardiol. 109, 63–70. 10.5935/abc.20170081 28678925 PMC5524477

[B52] Santos S. O. Loureiro S. M. A. Alves I. G. N. Jesus C. S. d. Santos P. R. d. Santos M. R. V. d. (2012). Experimental Gestational Hypothyroidism Evokes Hypertension in Adult Offspring Rats. Auton. Neurosci. 170, 36–41. 10.1016/j.autneu.2012.07.004 22878215

[B53] Silva R. B. Mesquita F. F. Andreo M. Assalin H. B. Rocha Gontijo J. A. Boer P. A. (2013). Effect of Gestational Protein Restriction on Left Ventricle Hypertrophy and Heart Angiotensin II Signaling Pathway in Adult Offspring Rats. Health 05, 78–84. 10.4236/health.2013.54a011

[B54] Silva A. S. Ariza D. Dias D. P. M. Crestani C. C. Martins-Pinge M. C. (2015). Cardiovascular and Autonomic Alterations in Rats with Parkinsonism Induced by 6-OHDA and Treated with L-DOPA. Life Sci. 127, 82–89. 10.1016/j.lfs.2015.01.032 25744393

[B55] Simas B. B. Nunes E. A. Crestani C. C. Speretta G. F. (2018). Cardiovascular and Metabolic Consequences of the Association between Chronic Stress and High-Fat Diet in Rats. Stress 21, 247–256. 10.1080/10253890.2018.1437413 29429380

[B56] Speretta G. F. Silva A. A. Vendramini R. C. Zanesco A. Delbin M. A. Menani J. V. (2016). Resistance Training Prevents the Cardiovascular Changes Caused by High-Fat Diet. Life Sci. 146, 154–162. 10.1016/j.lfs.2016.01.011 26776833

[B57] Valenti V. E. Ferreira C. Meneghini A. Ferreira M. Murad N. Ferreira Filho C. (2009). Evaluation of Baroreflex Function in Young Spontaneously Hypertensive Rats. Arq Bras Cardiol. 92, 205–215. 10.1590/s0066-782x2009000300009 19390709

[B58] Valenti V. E. De Abreu L. C. Colombari E. Sato M. A. Ferreira C. (2011). The Variability of Baroreflex Sensitivity in Juvenile, Spontaneously Hypertensive Rats. CardioVascular J. Africa 22, 14–17. 10.5830/cvja-2010-007 PMC4986285 21298200

[B59] WHO (2021a). Cardiovascular Diseases (CVDs). Fact sheet, 11 June 2021. Available at: https://www.who.int/news-room/fact-sheets/detail/cardiovascular-diseases-(cvds) (Accessed February, , 2022).

[B60] WHO (2021b). Hypertension. Fact sheet, 25 August 2021. Available at: https://www.who.int/news-room/fact-sheets/detail/hypertension (Accessed February, 2022).

